# Orientation of the Cochlea From a Surgeon’s Perspective

**DOI:** 10.1097/ONO.0000000000000017

**Published:** 2022-08-05

**Authors:** Nozomu Matsumoto, Nana Akagi-Tsuchihashi, Teppei Noda, Noritaka Komune, Takashi Nakagawa

**Affiliations:** Department of Otorhinolaryngology, Graduate School of Medical Sciences, Kyushu University, Fukuoka, Japan.

**Keywords:** Coiling plane, Misalignment, Tip fold-over

## Abstract

**Background:**

One of the mechanisms that cause tip fold-over is a misalignment between the electrode array’s coiling direction and the cochlea’s curving direction.

**Objectives:**

We reviewed surgical videos and computed tomography (CT) datasets of the patients who underwent cochlear implantation procedures from January 2010 to December 2021, paying particular attention to the cochlea’s orientation in the surgeon’s microscopic view.

**Methods:**

CT dataset and video recordings were analyzed to measure the “slope angle,” which is the angle between the cochlea’s coiling plane and the horizontal plane.

**Results:**

There were 220 cases that met the criteria and completed the analysis. The mean slope angle was 12.1° ± 9.5°, with a minimum of −9.4° and maximum of 44.6°. However, each surgeon had a favored slope angle range.

**Conclusion:**

Understanding the slope angle and making an effort to reduce the chance of misalignment during electrode insertion may help prevent tip fold-over of slim perimodiolar electrode arrays.

Cochlear implantation (CI) is an established surgical procedure, wherein a surgeon inserts a flexible electrode array into the scala tympani of the cochlea. There are 2 types of electrode arrays, namely a straight electrode array that curves passively at the lateral wall of the cochlea, and a precurved and perimodiolar electrode array that “hugs” the modiolus of the cochlea. To retain anatomical integrity within the cochlea and help preserve residual hearing, softer electrode arrays have been developed in both straight and perimodiolar electrode arrays.

Among these recently developed electrode arrays, soft perimodiolar electrode arrays are prone to bending or folding within the cochlea, a condition which is known as tip fold-over (1,2). Tip fold-over is not readily detectable during standard CI procedure and may result in some electrode contacts being switched off after device hook-up (3). Intraoperative radiographical (4) and electrophysiological (5) verification of the electrode placement helps address the tip fold-over before the patient leaves the operating room. However, given the intracochlear trauma caused by a bent electrode tip and multiple electrode insertion attempts, preventing the issue altogether is better than trying to cure it. One of the mechanisms that cause tip fold-over is a misalignment between the electrode array’s coiling direction and the cochlea’s curving direction (6). Thus, surgeons should take care regarding how they hold the soft perimodiolar electrode array during insertion and ensure that the electrode array’s coiling direction matches that of the cochlea. However, because the cochlea is concealed within the bone, successful alignment of the electrode array’s coiling direction and the cochlea’s curving direction largely depends on the surgeon’s correct imagination.

In the present study, we define 3 terms that are not commonly discussed elsewhere. First, the “coiling plane of the electrode array” was defined by the structural aspect of the perimodiolar electrode arrays (Fig. 1A). Straight electrode arrays can be curved in any direction and thus do not have specific coiling planes. The coiling plane of the electrode array is indicated on the device, for example, as a fin-shaped sheath handle of the CI632 electrode array (Cochlear, Macquarie Park, Australia). Second, the “coiling plane of the cochlea” is an imaginary plane defined by connecting the most inferior (caudal) point at the basal turn, the most superior (cranial) point at the basal turn, and the most anterior point in between these 2 points in the basal turn (Fig. 1B). Third, in the surgical microscopic view, the “slope angle” is defined as the angle between the horizontal plane and the coiling plane of the cochlea (Fig. 1C). Positive values in the slope angle indicate that the cochlea is climbing away from the ground as it coils. Theoretically, the surgeon’s task during electrode insertion is to align the coiling planes of the electrode array and the cochlea by holding the fin-shaped sheath handle (in cases of CI632) set at the slope angle. Note that this study is not about the “trajectory” of the insertion angle, that is, the direction from the round window to the basal turn of the cochlea (Fig. 1B). This study is about where the “fin” is facing at, under the condition that the surgeon is inserting the electrode array on the correct trajectory to the basal turn of the cochlea.

**FIG. 1.**
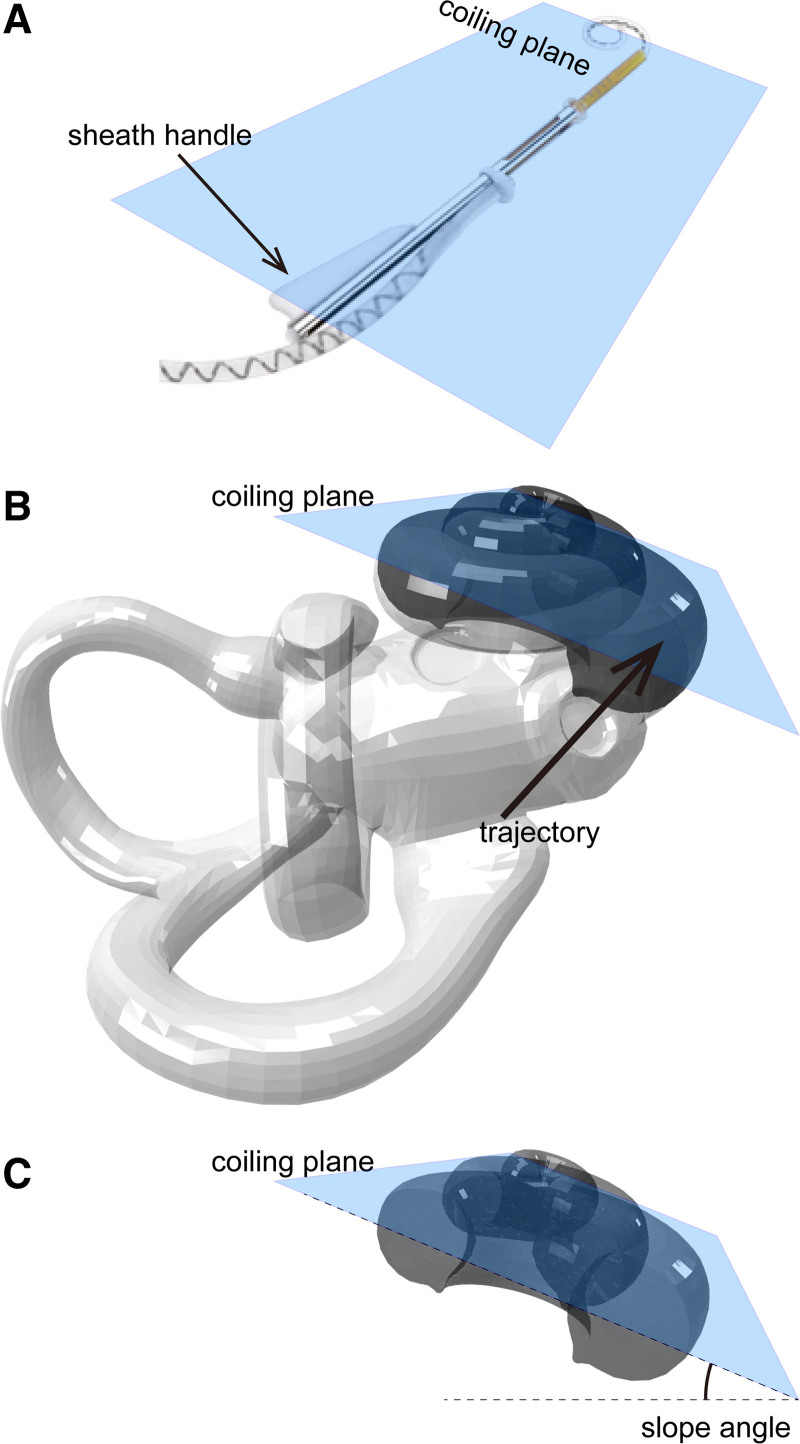
Coiling planes and the slope angle. *A*, The coiling plane of the electrode array. The CI632 device is used as an example, pictured here. The coiling plane and coiling direction of the precurved electrode tip are indicated on the device, for example, as the fin-shaped sheath handle on the CI632. *B*, The coiling plane of the cochlea. The anterior half of the right cochlea is drawn in dark gray, and the rest of the inner ear is drawn transparently. The coiling plane of the cochlea is defined by the caudal, cranial, and anterior end of the basal turn of the cochlea. *C*, The anterior half of the right cochlea. Angle between the coiling plane and the horizontal line from the surgeon’s view is defined as the slope angle. Note that this study is not about the “trajectory” of the insertion angle as shown in *B*, but about where the electrode’s coiling plane is facing at, under the condition that the surgeon is inserting the electrode array on the correct trajectory to the basal turn of the cochlea.

We found that there is surprisingly marked variation in the ways that surgeons hold the electrode array. On YouTube, where one can find instructional videos on CI performed by experts in this field, one surgeon held the electrode array so that it curved vertically down (slope angle of −90°), while another surgeon held it curving vertically up and even beyond (slope angle of >90°), with other orientations scattered in between (Fig. 2). A surgeon who prefers larger pillows may tilt the patient’s head steeper, thus increasing the surgeon’s typical slope angle. A surgeon who prefers having a bolster under the patient’s shoulder may decrease the typical slope angle. A surgeon who wishes to ensure that the electrode tip does not scratch the basal membrane would tilt the electrode’s coiling plane downward, intentionally misaligning the coiling planes on the safer side. Even when these surgeon’s individual preferences are taken into account, it is impossible to explain the wide variation of >180° in the slope angle in seemingly usual CI procedures. Thus, the variation in the electrode array’s coiling plane in instructional videos found on the Internet suggest substantial variation in the surgeons’ imaginary view of the cochlea.

**FIG. 2.**
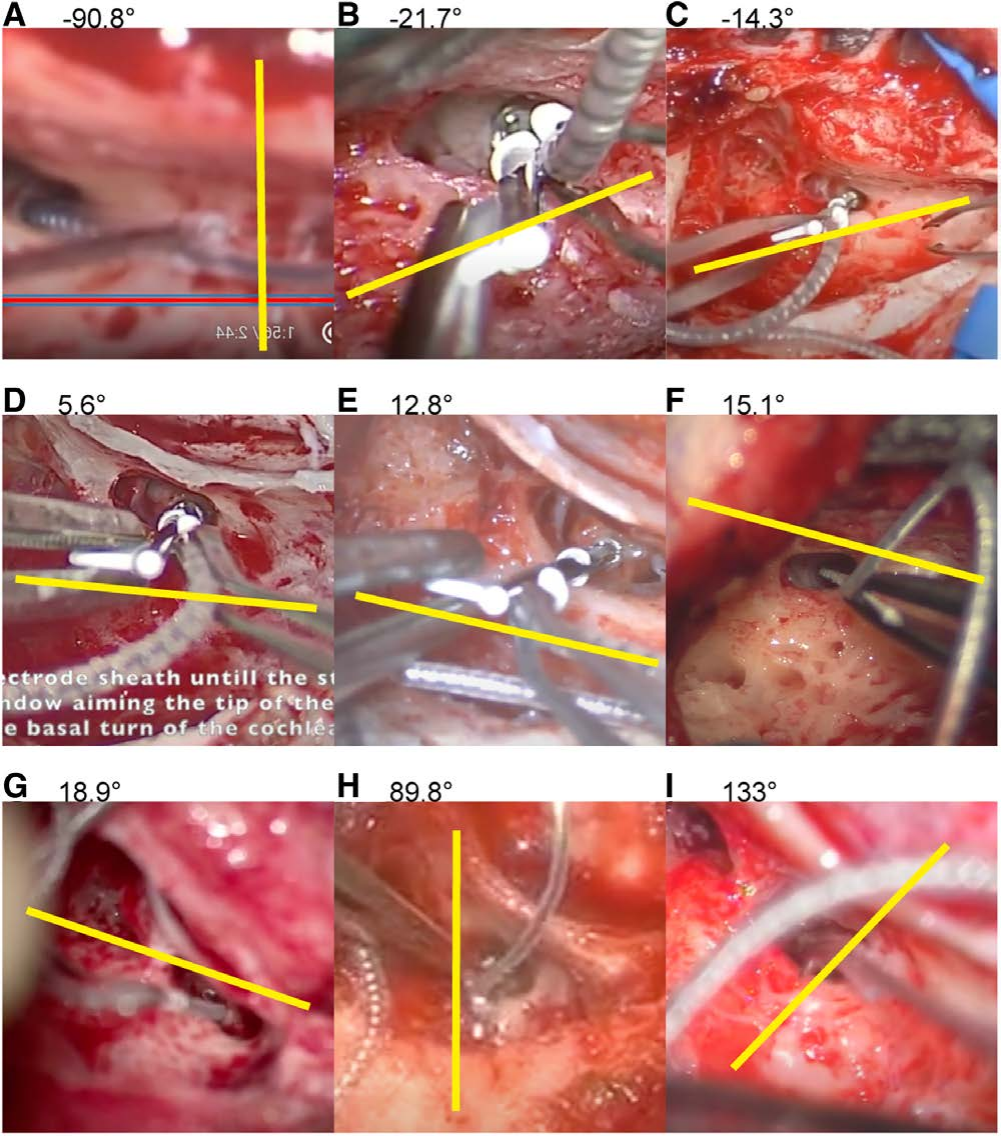
Surgeon’s orientation of the perimodiolar electrode arrays in videos available on the Internet. Images of the left temporal bones are flipped so that all images appear as the right side. Yellow lines indicate the direction of the electrode array’s coiling plane, judged by the directions of the sheath handle or stylet. Numerals on each panel indicate the angle of the yellow line from the horizontal line. All videos are freely available on the Internet. *A*, www.youtube.com/watch?v=iAV63jXQkXY. *B*, www.youtube.com/watch?v=claiXaVxUdE. *C*, www.youtube.com/watch?v=CM_cN1zzIW8. *D*, www.youtube.com/watch?v=9V1yAtqAl3c. *E*, www.youtube.com/watch?v=314S8auXk4k. *F*, www.youtube.com/watch?v=E0LHg2-OXG0. *G*, www.youtube.com/watch?v=gaqD2pVvbNQ. *H*, www.youtube.com/watch?v=WMe3yr2ZnUI. *I*, www.youtube.com/watch?v=A1XFz-ixTkE.

Through our previous research projects on virtual and augmented reality interfaces for otological procedures (7), particularly in image overlay cases (8), we have learned that the cochlea’s coiling direction is usually tilted slightly up from the horizontal plane (Fig. 3). Based on our experience, we routinely hold the electrode array so that its coiling plane is tilted about 10° up from the horizontal plane (slope angle of 10°). However, due to the small sample size and difficulty preparing augmented reality equipment for average daily cases, whether or not this “10° rule” applies to other cases or other surgeon’s cases is unclear.

**FIG. 3.**
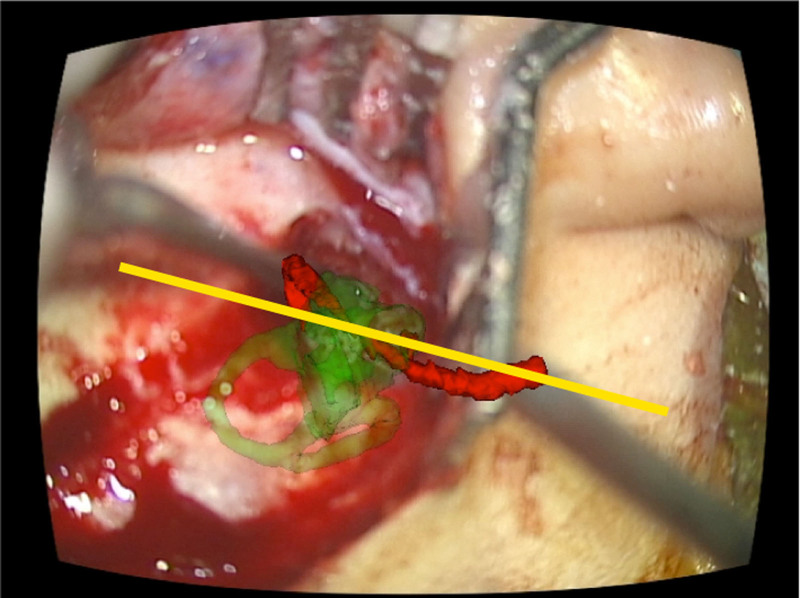
Snapshot of cochlear implantation assisted by the augmented reality interface. The image was flipped so that it appears as the right side. Green: Cochlea and semicircular canals, red: facial nerve. Yellow line indicates the coiling plane of the cochlea from the surgeon’s perspective. The slope angle was 14.9° in this case.

Therefore, we retrospectively investigated surgical videos and computed tomography (CT) datasets from our facility, paying particular attention to the cochlea’s orientation in the surgeon’s microscopic view.

## MATERIALS AND METHODS

This study was approved by the Institutional Review Board for Clinical Research of Kyushu University in 2022 as an opt out-approached research project (approval number: 22012-00).

### Patient Selection

CT datasets and video recordings of CI procedures performed from January 2010 to December 2021 were collected according to the following criteria: 1) complete preoperative CT dataset of temporal bone in digital format is available, 2) video contains information regarding the orientation of the temporal bone anatomy, and 3) surgeon who performed the operation is available for consultation. Exclusion criteria were as follows: 1) revision cases, 2) cases of malformation in the middle ear or cochlea, 3) cases that required procedures other than standard CI (eg, complete petrosectomy with blind-sac closure of the external ear canal), and 4) cases involving patients who opted out of the study. Bilateral cases were treated as 2 separate cases.

### Analyses

The preoperative CT dataset was 3-dimensionally reconstructed using the Aquarius iNtuition software program (Japanese version; TeraRecon, Tokyo, Japan) installed in the electronic medical chart system of the Kyushu University Hospital. Not all CT procedures were performed at Kyushu University Hospital. The CT resolution ranged from 0.19 to 0.45 mm in horizontal pixel size and 0.5 to 1.0 mm in slice pitch, based on the institutional CT protocol at the time that CT was performed.

**FIG. 4.**
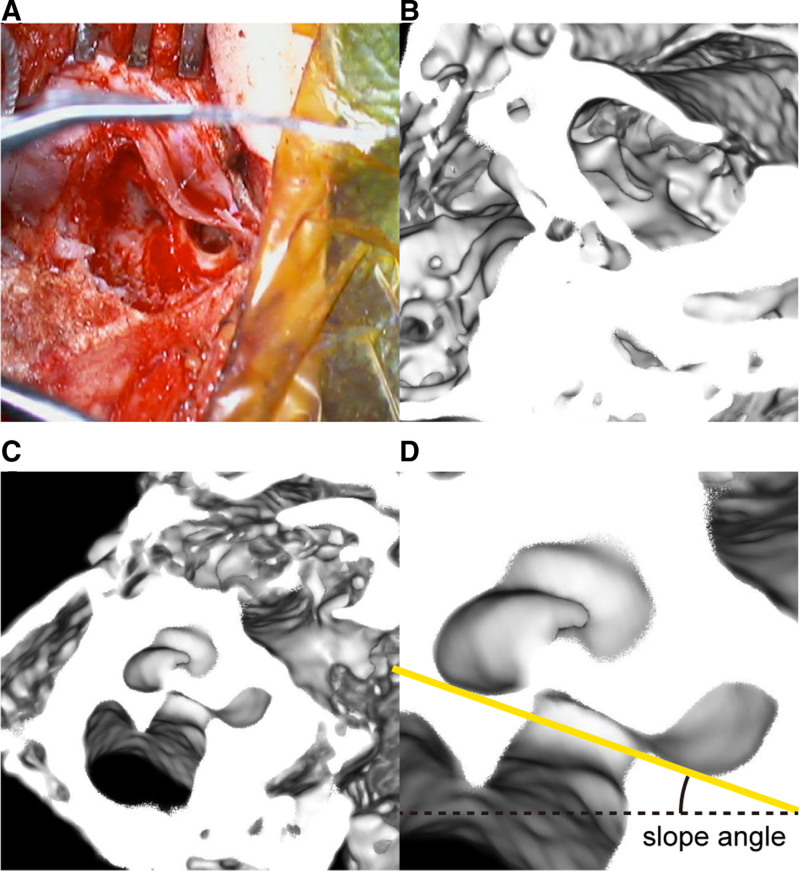
Measurement of the slope angle. *A*, In the video recording of the surgery just before electrode insertion, the locations and orientations of the anatomical structures in the temporal bone were identified. *B*, The 3D-reconstructed CT image is rotated to best describe the microscopic line of sight of *A*. *C*, The plane of the slab function, which is perpendicular to the line of sight, is moved so that it cuts the modiolus. *D*, The enlarged image of the anterior half of the cochlea. The yellow line connects the floors of the inferior end and the superior end of the basal turn. The “slope angle,” which is the angle between the yellow line and the horizontal line, was 19.4° in this case. CT indicates computed tomography.

We rotated the reconstructed volume image of the temporal bone to reproduce the orientation of the bone during the CI procedure as recorded in the video. A slicing function at the plane perpendicular to the viewing angle, along with the thick slab function, helped adjust the orientation using the images of the incus, stapes, round window, facial nerve, and other nonspecific bony features in each temporal bone. Each operating surgeon joined this analysis and approved the final orientation of the temporal bone just before electrode insertion. We then shifted the slicing plane to cut the modiolus of the cochlea. A straight line was defined on this slicing plane connecting the caudal side and cranial side of the basal turn of the cochlea. The line indicated the coiling plane of the cochlea, and the angle between this line and the horizontal line in degrees (slope angle) was collected for the analysis (Fig. 4).

Other collected information included the operated date, the operated side, and the patient’s age at the time of operation. Four surgeons were involved in the analyzed surgery, and all were right-handed.

Numerals were described as the mean ± standard deviation. A non-paired, 2-tailed Student’s *t* test was used for the statistical analyses. Differences with *P* < 0.05 were judged as statistically significant.

## RESULTS

There were 220 cases that met the criteria and completed the analysis. The number of analyzed cases for surgeons A, B, C, and D were 165, 18, 19, and 18 CI procedures, respectively. The mean slope angle was 12.1° ± 9.5°, with a minimum of −9.4° and maximum of 44.6°.

**FIG. 5.**
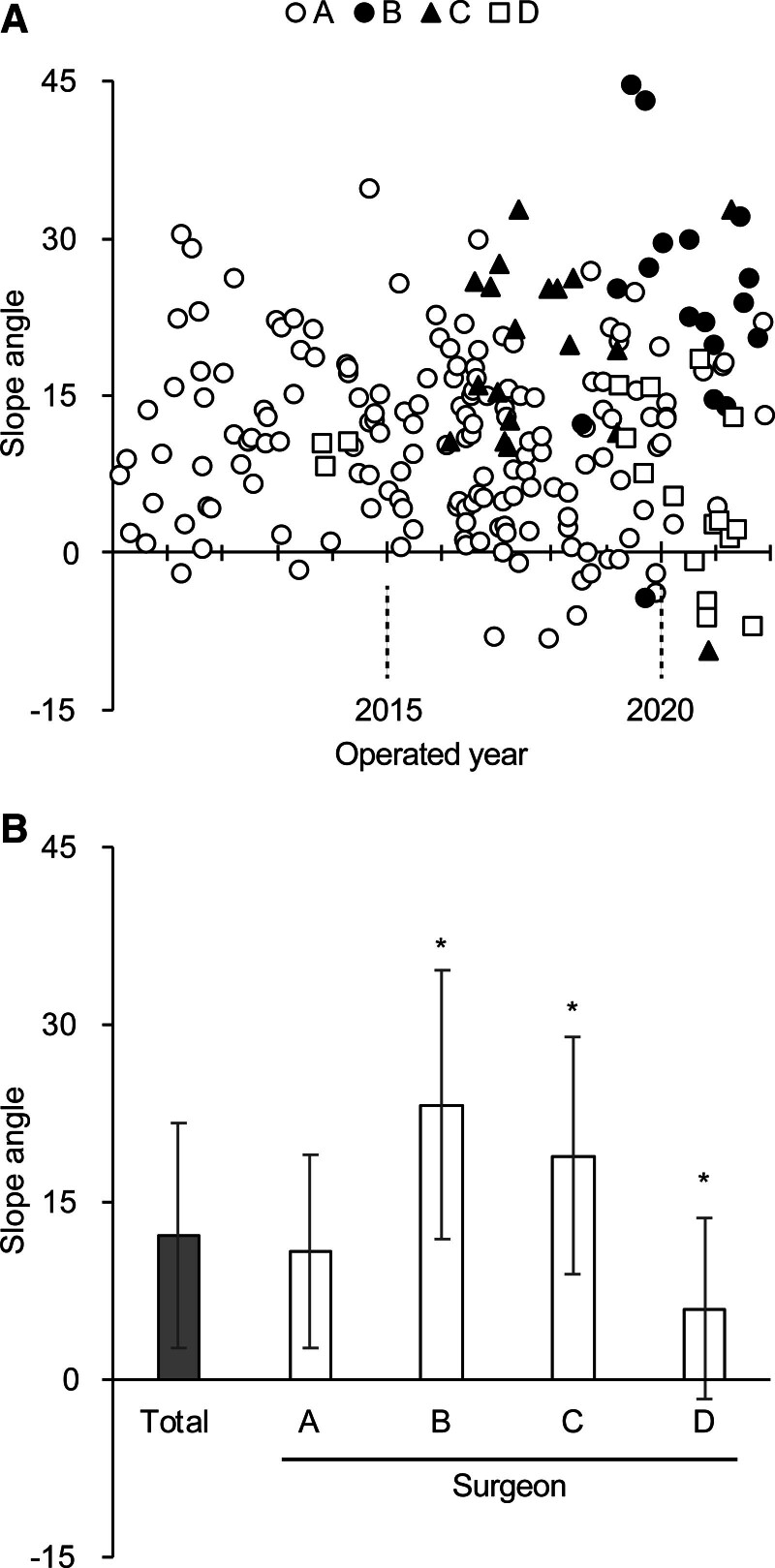
The slope angles of the cochlea in each surgeons’ line of sight. *A*, Each marker represents the slope angle measured in each surgery. *B*, Each column and error bars represent the mean and SD of the slope angles, respectively. Asterisks indicate statistically significant differences compared with the slope angle of surgeon A’s cases.

We also found that each surgeon had a favored slope angle range. Surgeon A, who was the most senior of the group, preferred 10.8° ± 8.2°. Conversely, surgeons B (23.2° ± 11.3°) and C (18.9° ± 10.0°) had larger preferred slope angles (statistically significant, *P* < 0.001, *P* < 0.001, respectively), while surgeon D (5.97° ± 7.7°) had a smaller preferred slope angle (statistically significant, *P* = 0.017; Fig. 5).

**FIG. 6.**
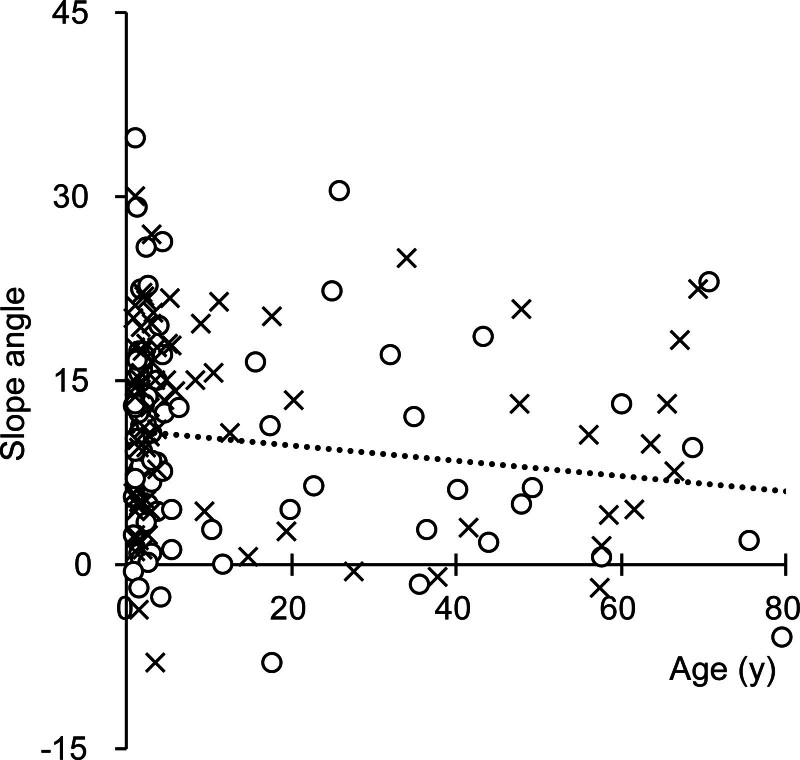
Operating side and the patient’s age. Circles: right ear, crosses: left ear. The dotted line indicates the linear regression, whose function was (slope angle) = −0.062 × (age) + 10.87.

Additional analyses were performed for surgeon A’s cases. The patient age at the time of surgery had a minimal effect on the slope angle. There was a trivial tendency for the slope angle to decrease with age: about 6° per 100 years of age. The side of the surgery had no marked impact on the slope angle. The CI on the right side had a 10.1° ± 8.2° slope angle, while that of the left side was 11.6° ± 8.1°, a not statistically significant difference (*P* = 0.23, Fig. 6). We did not note any learning effect on the slope angle. The slope angles of surgeon A’s earliest 30 cases (11.5° ± 8.3°) and most recent 30 cases (11.7° ± 8.0°) did not show a statistically significant difference (*P* = 0.96).

## DISCUSSION

Precurved, perimodiolar electrode arrays have their specific coiling direction defined by the physical property of the material. Even if a surgeon correctly places the electrode array in the scala tympani through correct trajectory, if the electrode array is rolled around the axis of the electrode array itself, it can cause misalignment of the coiling planes of the electrode array and the cochlea (Fig. 1). Misalignment of the perimodiolar electrode array during insertion may result in the tip bending the wrong direction within the cochlea, potentially injuring the intracochlear structures (9), and may cause tip fold-over or scalar translocation (2). Because the electrode array is completely passive, tip fold-over occurs only when there is enough force applied to the tip of the electrode array, such as bumping onto the wall of the cochlea. Tip fold-over can be intentionally caused by misaligning the electrode array upon insertion (6). Therefore, careful alignment of the electrode array’s coiling plane and the cochlea’s coiling plane deserves close attention when dealing with slim perimodiolar electrode arrays. Note that this study is not about the “trajectory” of the insertion angle, but about where the electrode’s coiling plane is facing at, under the condition that the surgeon is inserting the electrode array on the correct trajectory to the basal turn of the cochlea.

Tip fold-over is rarer in straight electrode arrays than in perimodiolar electrode arrays (10,11), but this does not mean that straight electrode arrays are free from issues caused by misalignment. Some straight electrode arrays have half-band electrode contacts, designating a specific side that should face the modiolus of the cochlea. Misalignment of these electrode arrays may increase the electrical distance between the spiral ganglion and the electrode contacts, resulting in suboptimal performance of the device. Thus, understanding the orientation of the cochlea is important in any electrode arrays, not just in perimodiolar electrode arrays.

How a surgeon holds the electrode array indicates the surgeon’s image of the cochlea. Numerous video lectures, teaching materials, presentations, and personal communications have demonstrated the huge variation in how the electrode array is held by surgeons upon insertion. Some surgeons hold the electrode array so that the tip coils horizontally, while others hold the array with a vertical coil. Obviously, misalignment of the coiling planes is not the only mechanism for the tip fold-over. Other known factors that may cause tip fold-over include inadequate visualization of the round window, rough handling of the electrode array, failure to insert electrode array slowly and steadily and so on. The importance of these known factors is already shared by virtually all CI surgeons. On the other hand, the variations that exist in the way that how surgeons hold the perimodiolar electrode arrays indicate that some CI surgeons are relatively unconcerned with aligning the electrode array’s coiling plane and the cochlea’s coiling plane. YouTube videos alone suggest that not all expert CI surgeons are aware of the coiling plane of the electrode array (Fig. 2). Thus, there is a lack of awareness in the coiling planes and the importance of aligning the planes. This fact may become an issue when the outcomes of straight and perimodiolar electrode arrays are statistically compared (12), as surgeons who do not take care to align the coiling planes may cause unfavorable outcomes more often in cases with perimodiolar electrode arrays than in those cases who chose straight electrode arrays. For example, the evaluation of hearing preservation outcomes in slim perimodiolar electrode arrays vary widely, with some studies with smaller group of surgeons finding such devices to be feasible (13,14), while a meta-analysis involving a wider range of surgeons in 33 studies recommend against the use of perimodiolar arrays (15).

Surgeon A learned through his research that the cochlea’s coiling plane is about 10° up from the horizontal plane and instructed other surgeons in the group to maintain this angle upon electrode insertion. Thus, all 4 surgeons in this study held the electrode array in the same way (tilted 10° up from the horizontal line) during electrode insertion. Our results indicated that this “10° rule” was only partly correct. The slope angle of the basal turn of the cochlea averaged at 12.1°, proving the 10° rule reasonable; however, our results also indicated that each surgeon had a typical slope angle they adopted. Furthermore, each surgeon’s favorite pillow, preferences concerning how to place the patient’s head, whether to use or remove a bolster under the patient’s shoulder, and other miscellaneous factors contributed to the differences in slope angles. Therefore, rather than dictate a certain slope angle be adopted by other surgeons, we should instead encourage them to review their most recent 10 to 20 surgeries to determine their typical slope angle.

The slope angles in surgeon A’s cases did not change markedly over time, so it is unlikely that a surgeon’s slope angle changes as the surgeon experiences more CI procedures. In addition, right-handed surgeons usually secure more room for the right hand in the operating field, so the slope angle may differ by the operating side for some surgeons. The analysis of surgeon A’s cases did not show a marked difference in the slope angle with the operating side, but this may have been because surgeon A held the electrode array in his left hand when operating on the right ear and switched hands for the left ear. Ultimately, each case can be preoperatively evaluated using a CT viewer software program in the electronic medical chart system capable of 3-dimensional reconstruction and reslicing in different planes. If such a software program is not installed in the electronic medical chart system, free software programs are available with even more sophisticated image analysis functions. This preoperative effort, which only takes about 10 minutes to perform, may reduce the chance of misalignment during electrode insertion and contribute to prevent the tip fold-over. As of the time this manuscript was submitted, none of the 4 surgeons in this study have experienced a single case of tip fold-over.

In conclusion, understanding the slope angle and making an effort to reduce the chance of misalignment during electrode insertion may help prevent tip fold-over of slim perimodiolar electrode arrays. In addition, understanding the orientation of the cochlea is important in any electrode arrays, not just in perimodiolar electrode arrays.

## FUNDING SOURCES

Part of this study was supported by the Japan Society for the Promotion of Science (JSPS) KAKENHI Grant Number 18K09380 to N. Matsumoto.

## CONFLICT OF INTEREST STATEMENT

T. Nakagawa and his entire team including all the authors hold a contracted research project funded by Cochlear Japan, and we declare this fact as a potential conflict of interest.

## DATA AVAILABILITY

The dataset generated and analyzed during the current study is not publicly available because it contains information that can potentially reveal patients’ privacy, but is available from the corresponding author on reasonable request.
